# Transcriptome Profiling Identifies Differentially Expressed Genes in Huoyan Goose Ovaries between the Laying Period and Ceased Period

**DOI:** 10.1371/journal.pone.0113211

**Published:** 2014-11-24

**Authors:** Xinhong Luan, Dawei Liu, Zhongzan Cao, Lina Luo, Mei Liu, Ming Gao, Xiaoying Zhang

**Affiliations:** 1 College of Animal Science and Veterinary Medicine, Shenyang Agricultural University, Shenyang, 110866, China; 2 Liaoning Province Livestock and Poultry Genetic Resources Conservation and Utilization Center, Liaoyang, 111000, China; University of Florida, United States of America

## Abstract

The Huoyan goose is famous for its high egg-laying performance and is listed as a nationally protected domestic animal by the Chinese government. To elucidate the key regulatory genes involved in Huoyan goose egg laying, RNA from ovarian tissue during the ceased and laying periods was sequenced using the Illumina HiSeq 2000 sequencing platform. More than 12 million reads were produced in ceased and laying libraries that included 11,896,423 and 12,534,799 clean reads, respectively. More than 20% of the reads were matched to the reference genome, and 23% of the reads were matched to reference genes. Genes with a false discovery rate (FDR) ≤0.001 and log_2_ratio ≧1 or ≤−1 were characterized as differentially expressed, and 344 up-regulated and 344 down-regulated genes were classified into functional categories. Twelve genes that are mainly involved in pathways for reproduction regulation, such as steroid hormone biosynthesis, GnRH signaling pathways, oocyte meiosis, progesterone-mediated oocyte maturation, steroid biosynthesis, calcium signaling pathways, and G-protein coupled receptor signaling pathway were selected for validation by a quantitative real-time polymerase chain reaction (qRT-PCR) analysis, the qRT-PCR results are consistent with the general expression patterns of those genes from the Illumina sequencing. These data provide comprehensive gene expression information at the transcriptional level that might increase our understanding of the Huoyan goose's reproductive biology.

## Introduction

The Huoyan goose is a native Chinese breed that tolerates coarse feed and cold temperatures, is extremely adaptable and weakly broody, and has high egg-laying rates and outstanding reproductive performance. It is considered a national treasure by the Chinese goose industry and was listed as one of the nationally protected domestic animals by the Chinese government in 2000 [1], [2]. But recently, problems of variety degeneration, especially a decrease in the number of eggs laid, have become prominent and are hindering the conservation and utilization of this local goose breed. In order to elucidate the molecular mechanism that controls egg-laying in geese, the reproductive biology of the Huoyan goose need to be investigated.

The number of eggs laid by a bird is determined by the number of follicles destined for ovulation and the capacity of the oviduct to transform the ova into a hard-shelled egg. Beside environment and metabolism factors, ovarian follicle growth and development are also controlled by a myriad of endocrine, paracrine and autocrine factors, including gonadotropins, sex steroid hormones and growth factors. In poultry, the reproductive endocrine system and reproductive activity are strictly controlled by the hypothalamic-pituitary-gonadal axis [3]. The hypothalamus regulates reproduction by releasing neurohormones (gonadotropin-releasing hormones, GnRH) to the pituitary gland. The pituitary gland then synthesizes and releases gonadotropins (luteinizing hormone, LH; follicle-stimulating hormone, FSH), which in turn act on the gonads to stimulate gametogenesis (spermatogenesis, oogenesis) and sex steroid hormone secretion (gestagens, androgens, and estrogens). The ovary performs numerous roles critical for oocyte development and ovulation. Within the ovary, granulose cells are an important site for estrogen production for local use as well as providing endocrine signaling to other tissues [4]. The somatic cells of the ovary act to support the growth and development of the oocyte in preparation for the surge of luteinizing hormone (LH) which elicits a physiological response leading to ovulation. This response includes promoting meiosis, steroidogenesis, follicular development, cumulus cell expansion, luteinization, and progesterone production, ultimately promoting oocyte maturation [5], [6]. The dynamic and highly regulated process of follicle development requires the coordinated actions of a great number of genes, which is orderly orchestrated at the transcriptional and posttranscriptional levels. Recently, genomic and transcriptomic studies have identified genes that are related to high egg production and expressed specifically in the hypothalamic-pituitary-gonadal axis (HPG). Shiue (2006) identified nine transcripts related to high egg production in the chicken hypothalamus/pituitary gland, thus providing a valuable resource for the identification of markers of high egg production in chickens [7]. Kang et al. (2009) identified 18 known and 8 unknown differentially expressed genes in the ovaries from the pre-laying to egg-laying stage [8]. Yen (2006) found that at least 19 genes were more highly expressed in the pituitary glands of laying geese compared with pre-laying geese [9]. Our team used the suppression subtractive hybridization (SSH) method to investigate gene expression profiles in the hypothalamus and pituitary gland of Huoyan geese during the laying period and ceased period [2], [10]. However, these limited genetic data for geese do not provide global transcriptome information and are insufficient for elucidating the molecular mechanisms of productivity in the laying goose.

In recent years, the development of next-generation sequencing (NGS), which is also known as high-throughput or deep sequencing technology, has provided a powerful, highly reproducible and cost-efficient tool for transcriptomic research [11]. An advantage of the technology is that it may be used for gene discovery and expression profiling of organisms without a reference genome by the *de novo* assembly of generated short reads [12], [13], [14]. There are several advanced and alternative NGS platforms, such as Roche's 454 GS FLX, Illumina HiSeq 2000 and Applied Biosystems' SOLiD [15].

In this study, high-throughput sequencing technology was applied to generate comprehensive transcriptomic profiles of ovarian tissue from Huoyan geese during the ceased and laying periods using the Illumina HiSeq 2000 sequencing platform. A comparative analysis of transcriptomic data was performed, and a large number of genes were found to be differentially expressed between the ceased and laying periods. Our analysis found that certain genes involved in pathways for reproduction regulation, such as steroid hormone biosynthesis, GnRH signaling pathways, oocyte meiosis, progesterone-mediated oocyte maturation, steroid biosynthesis, calcium signaling pathways, G-protein coupled receptor signaling pathway, dopaminergic synapse, and MAPK signaling pathway are differentially regulated. Additionally, expression profiling of these differentially regulated genes were validated by quantitative real-time polymerase chain reaction (qRT-PCR).

## Materials and Methods

### Ethics statement

Experimental procedures were approved by the animal welfare committee of the College of Animal Science and Veterinary Medicine of Shenyang Agricultural University (No. 2011036) and performed in accordance with the Regulations for the Administration of Affairs Concerning Experimental Animals (China, 1988) and EU Directive 2010/63/EU for animal experiments. All of the surgery was performed according to recommendations proposed by the European Commission (1997), and all efforts were made to minimize the suffering of the animals.

### Experimental animal and sample preparation

The Huoyan geese were selected from the Liaoning Huoyan Goose Stock Breeding Farm and raised according to the program used at the farm. During the experiment, geese were fed ad libitum with rice grain and supplemented with green grass or water plants whenever possible. Feed was provided during the daytime when the geese were released into an open area outside of the building. Nine peak-laying period geese were sampled at 12 months of age (average BW = 3.5±0.6 kg). Nine ceased period geese were sampled at 15 months of age (average BW = 3.5±0.4 kg). Geese were killed by exsanguination to obtain ovary samples, which comprised the whole ovary including the small and large yellow follicles. For those ovarian follicles at different development stages, the walls of ovarian follicles were isolated for RNA extraction. All of the samples were quickly dissected, frozen in liquid nitrogen, and stored at −80°C. Numbers of eggs to sampling day were recorded for each goose in laying period group and ceased period group from the day of first egg to sampling day. Their average eggs to sampling day (mean ± S.E.M.) were 83.7±2.3 and 115.3±6.6, respectively.

### RNA extraction, library preparation and sequencing

The total RNA was extracted using TRIzol reagent (Invitrogen, USA) following the manufacturer's protocol. The RNA quality was characterized initially on an agarose gel and NanoDrop 8000 spectrophotometer (NanoDrop, Thermo Scientific), and the integrity of the RNA samples was evaluated using a 2100 Bioanalyzer (Agilent Technologies, USA). To minimize the effect of transcriptome unevenness among individuals, the RNA samples from nine individuals were pooled within each group in equal amounts to generate one mixed sample per group. These mixed RNA samples were subsequently used to construct a complementary DNA (cDNA) library and perform Illumina deep sequencing.

The cDNA libraries of the ceased period and laying period were constructed according to the Illumina kit's manufacturer's instructions (Illumina, San Diego, CA). Briefly, a fragmentation buffer was mixed with magnetic beads and Oligo(dT) was used to isolate the messenger RNA (mRNA), and then the mRNA was fragmented into shorter fragments. The first strand of cDNA was then synthesized with random hexamer-primer using the mRNA fragments as templates. Buffer, deoxynucleotide triphosphates (dNTPs), Ribonuclease H (RNase H) and DNA polymerase I were added to synthesize the second strand. Double-stranded cDNAs were purified with the QiaQuick PCR extraction kit (Qiagen, Germany) and eluted with EB buffer for end repair and poly(A) addition. Finally, sequencing adapters were ligated to the 5′ and 3′ ends of the fragments, and the fragments were purified by agarose gel electrophoresis and enriched by PCR amplification to create a cDNA library. During the quality control (QC) steps, the Agilent 2100 Bioanalyzer and the ABI StepOnePlus Real-Time PCR System were used for quantification and qualification of the sample library. Finally, the library was sequenced using an Illumina HiSeq 2000 at the Beijing Genomics Institute (BGI) in Shenzhen, China. All of the technical steps were performed in duplicate.

### Sequence data analysis and assembly

The original image data were translated into sequence data as raw data or raw reads via base calling. Because the algorithms used in *de novo* transcriptome construction of the short reads provided by the Illumina platform may be severely inhibited by sequencing errors, a stringent filtering process of the cDNA sequence was employed to select only clean reads. This process including the removal of reads with adaptors, reads with more than 5% unknown nucleotides and low-quality reads in which the percentage of low-quality bases (base quality ≤5) was higher than 50%. An analysis was then performed of the clean read data, which included the sequence quality assessment, saturation analysis of sequencing, experimental repeatability analysis, distribution of clean reads copy number, and alignment statistics of clean reads.

### Reads mapping to reference genome and genes

Because of a lack of genomic resources for geese, an essential dataset containing reference genes expressed in the chicken genome was prepared to identify the genes corresponding to the reads in each library. The chicken reference genome and gene sets were downloaded from the official website of the National Center of Genome Research (ftp://ftp.ncbi.nih.gov/genomes/Gallus_gallus, ftp://ftp.ncbi.nih.gov/genomes/Gallus_gallus/Assembled_chromosomes/). All of the clean reads were mapped to the chicken genome using the Short Oligonucleotide Analysis Package alignment tool SOAPaligner/SOAP2 [16], which does not allow mismatches of more than two nucleotides.

### Identification of differentially expressed genes

To identify the differentially expressed genes between the ceased period and laying period, expression levels were calculated using the RPKM (Reads Per Kb per Million mapped reads) method as described by Mortazavi et al. [17]. Differentially expressed genes (DEGs) were identified using a rigorous algorithm developed by BGI based on the method described by Audic et al. [18]. The false discovery rate (FDR) was used to determine the threshold *P*-Values in the multiple tests and analyses through manipulation of the FDR values. We used *P*≤0.01, FDR ≤0.001 and |log_2_ratio| ≧1 as the threshold to judge the significance of gene expression differences [19].

For gene ontology (GO) annotations and GO functional classifications of DEGs, the Blast2GO program was used [20]. A gene ontology enrichment analysis and Kyoto Encyclopedia of Genes and Genomes (KEGG) pathway enrichment analysis were performed to identify which DEGs were significantly enriched in GO terms and metabolic pathways at the Bonferroni-corrected *P*-value of ≤0.05 compared with the whole-transcriptome background using the formula described by Ting Shi et al. [21].

### Quantitative Real-time PCR (qRT-PCR) Validation

To validate the reliability of the Illumina analysis, twelve putative genes involved in the regulation of reproduction and reproductive processes and sex steroid hormone biosynthesis were selected and detected by qRT-PCR. The qRT-PCR primers were designed using Primer 3.0 (http://frodo.wi.mit.edu/primer3), and those used for qRT-PCR analysis are listed in Table S1. Tissues from every three birds were pooled in each group, and then there were three sample pools in each group. The total RNA was extracted as described for the transcriptome library preparation and sequencing.

Two micrograms of total RNA was reverse-transcribed using the PrimerScript RT Reagent Kit (TaKaRa, Dalian, China), and the RT-PCR analysis was conducted on the Bio-Rad iQ5 Real-time PCR Detection System (BIO-RAD, California, USA). Each 25 µl reaction volume contained 1 µl 10 µM (each) forward and reverse primers, 12.5 µl 2× SYBR Premix Ex Taq II (Takara, Dalian, China), and 2 µl cDNA products, and the final volume was adjusted using PCR-water. The following PCR program was used for amplification: 15 min at 95°C, 40 cycles of denaturation at 95°C for 10 s and annealing and extension at 60°C for 30 s. Beta-actin (ACTB) was selected as an internal reference gene, and the expression level of ACTB was used to normalize the qRT-PCR results for each gene. Negative controls without the cDNA template were included in this experiment. The standard curve testing was performed using a series of 10-fold diluted samples for the different genes. The slopes of standard curves and PCR efficiency of these genes were calculated to confirm if the qRT-PCR data were precise and reliable. The melting curves were analyzed to make sure that a single PCR product was amplified for each pair of primers, and the expression levels of these genes were verified from three independent biological replicates along with the internal reference gene. Fold changes were calculated using the 2^−ΔΔCt^ method [22]. All of the data were expressed as the mean ± standard deviation, and a statistical analysis using Student's t tests was performed with SPSS 16.0 for Windows (SPSS Inc.) to evaluate whether the means were significantly different (*P*<0.05) or highly significantly different (*P*<0.01).

### Accession code

The Illumina HiSeq 2000 sequencing data for the goose ovary transcriptome have been deposited in the National Center for Biotechnology Information (NCBI) Sequence Read Archive (SRA, http://www.ncbi.nlm.nih.gov/Traces/sra) with the accession number SRR1119186.

## Results

### Illumina transcriptome sequencing and reads assembly

**Figure 1:**
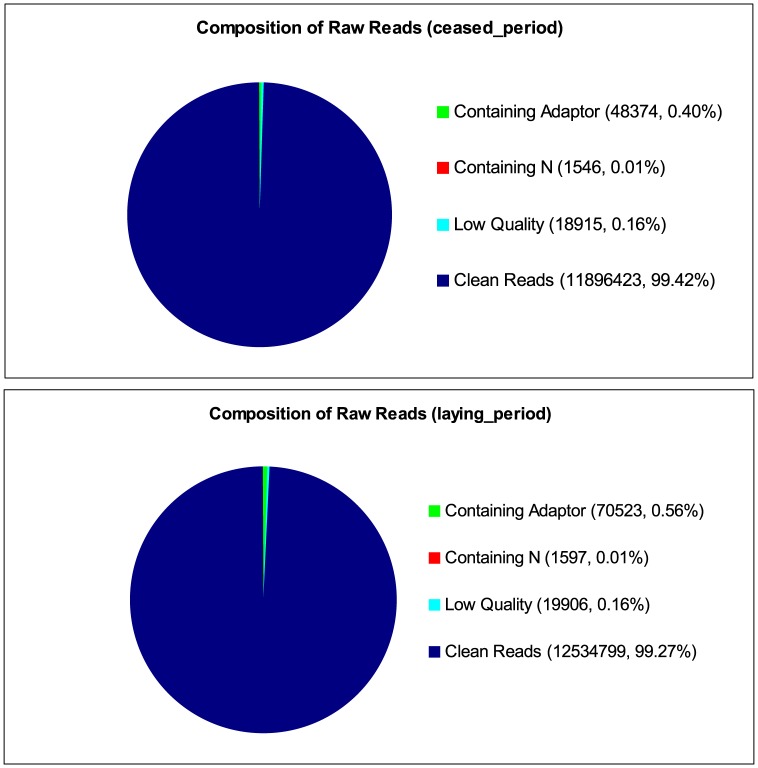
Different components of the raw reads in each sample. The percentages of reads containing N, adaptors, clean reads and low-quality reads. The numbers in parentheses indicate the percentage of each type of read among the total raw reads.

To obtain a global view of the goose ovary transcriptome and identify the genes involved in the regulation of egg-laying, cDNA libraries from the ovarian tissues of ceased period geese and laying period tissues were constructed and sequenced using the Illumina HiSeq 2000 sequencing platform. More than 11.9 million and 12.6 million raw tags were generated in each library. Approximately 99% of the raw reads passed the filter, with 11,896,423 and 12,534,799 clean reads acquired in the two libraries (Figure 1). The total produced base pairs were 582,924,727 and 614,205,151 in each library (Table 1).

**Table 1:** Alignment statistical results of the ceased_period library and laying_period library.

Sample	Ceased_period		laying_period	
Total Reads	11896423		12534799	
Total Base Pairs	582924727		614205151	
**Map to Genome**	**reads number**	**percentage**	**reads number**	**percentage**
Total Mapped Reads	2200951	18.50%	2515481	20.07%
perfect match	379968	3.19%	416772	3.32%
< = 3 bp mismatch	1820983	15.31%	2098709	16.74%
unique match	2118775	17.81%	2434981	19.43%
multi-position match	82176	0.69%	80500	0.64%
Total Unmapped Reads	9695472	81.50%	10019318	79.93%
**Map to Gene**				
Total Mapped Reads	2462292	20.70%	2941957	23.47%
perfect match	371746	3.12%	432179	3.45%
< = 3 bp mismatch	2090546	17.57%	2509778	20.02%
unique match	1713631	14.40%	2006440	16.01%
multi-position match	748661	6.29%	935517	7.46%
Total Unmapped Reads	9434131	79.30%	9592842	76.53%

### Assessment of cDNA Libraries

**Figure 2:**
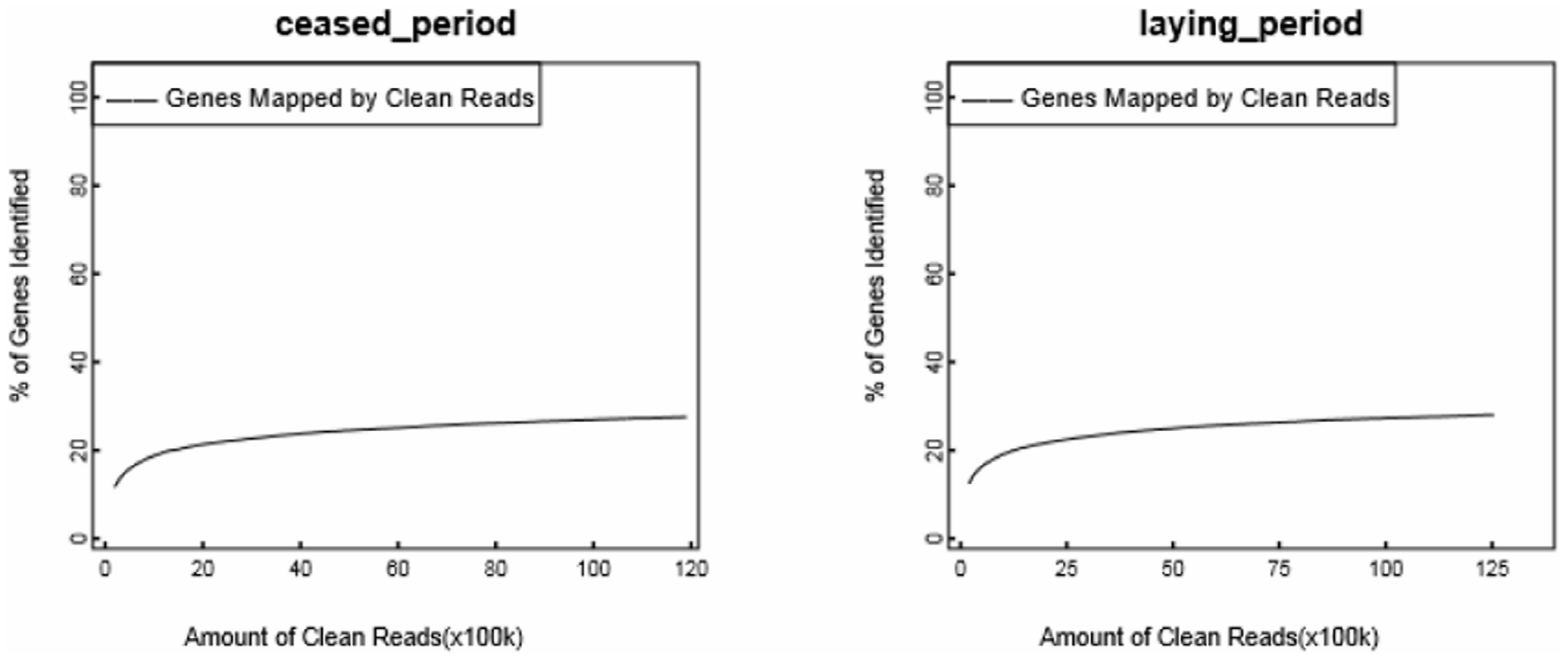
Sequencing saturation analysis. The X-axis is the number of clean reads, and the Y-axis is the percentage of identified genes. When the number of reads reaches 5 M, the growth rate of the detected genes flattens, indicating that the number of detected genes tends towards saturation. The left figure shows the DGE of the ceased-period goose, and the right figure shows the DGE of the laying-period goose.

To confirm that the number of detected genes increases proportionally to the sequencing amount, a saturation analysis was performed. A sequence saturation analysis is used to measure the sequencing data of a sample. With an increasing number of reads, the number of detected genes increases as well. However, when the number of reads reaches a certain value, the growth rate of the detected genes flattens and saturates. Figure 2 shows a trend of saturation where the number of detected genes almost ceases to increase when the number of reads reaches approximately 5 million.

**Figure 3:**
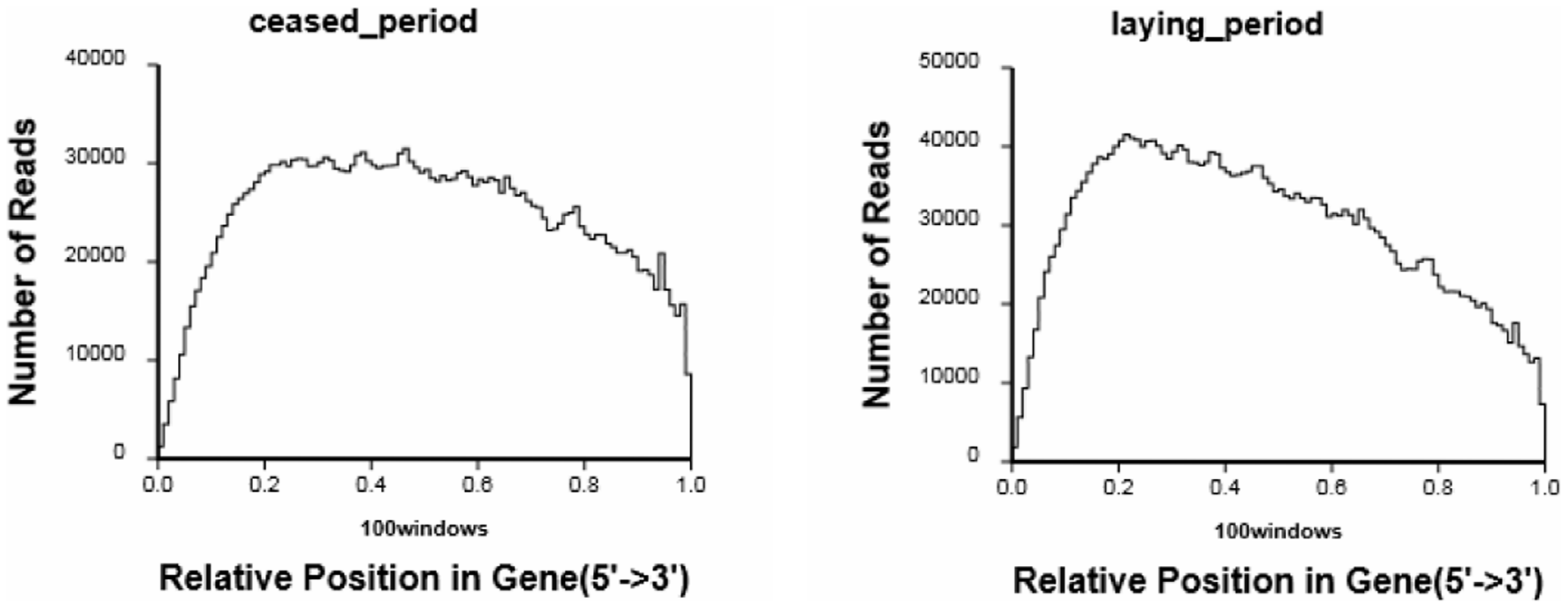
Distribution statistics of reads mapped to reference genes. The horizontal axis represents the relative read position, which is calculated as the ratio between a read's location on the gene and the gene length, and the vertical axis represents the numbers of reads that are mapped to genes.

During preparation of the cDNA sequencing libraries, the mRNA was first fragmented into short segments by chemical methods and then sequenced. If the randomness of breaking is poor, the read preference from a specific gene region will directly affect the subsequent analysis. We used the distribution of read location on the genes to evaluate the randomness of breaking. Because genes have different lengths, the read location on a gene was standardized to a relative position (which was calculated as the ratio between the reads location on the gene and gene length), and then the number of reads in each position was counted [23]. In this study, the evenly distributed reads in every position of the genes indicated that the randomness of breaking of these two samples is good (Figure 3).

### Reads mapping to the reference genome dataset

To identify the genes corresponding to clean reads in each library, the clean reads were mapped to the reference genes expressed in the chick genome. The mapping results showed that 3.19% (379,968) and 3.32% (416,772) of the reads from each library were perfectly matched to the reference genome, whereas approximately 3.12% (371,746) and 3.45% (432,179) of the reads from each library were perfectly matched to the reference genes. The percentages of unique reads matched to the genome were 17.81% (2,118,775) and 19.43% (2,434,981) and the percentages matched to the reference genes were 14.40% (1,713,631) and 16.01% (2,006,440) in the two libraries. Altogether, there were 18.50% (2,200,951) and 20.07% (2,515,481) reads matched to the reference genome and 20.70% (2,462,292) and 23.47% (2,941,957) reads matched to the reference genes. The alignment statistics of these reads from the ceased period and laying period libraries are shown in Table 1.

**Figure 4:**
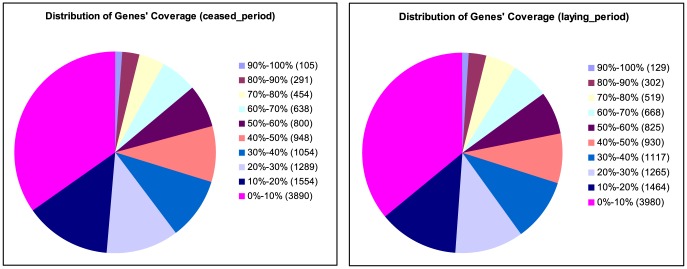
Distribution of gene coverage identification for differentially expressed genes. Gene coverage is used to assess the depth of DGE sequencing and refers to the percentage mapped by reads of each gene.

Gene coverage was calculated as the percentage of a gene covered by reads from each of the ceased period and laying period libraries. Figure 4 shows the distribution gene coverage of the two samples. Approximately 1% of the total genes of the ceased and laying period samples had coverage between 90–100% (105 and 129 genes, respectively), and 3% of the total genes of the ceased and laying period samples had coverage between 80–90% (291 and 302, respectively) respectively.

### Analysis of gene differential expression

**Figure 5:**
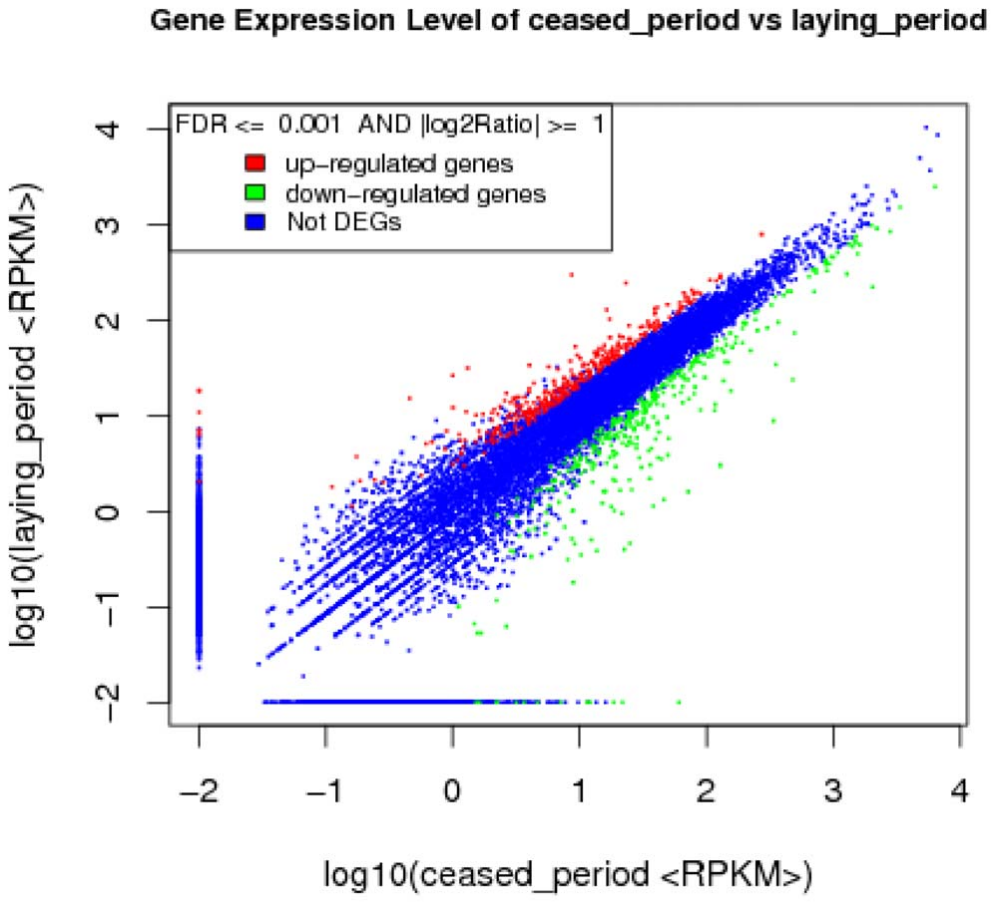
Comparison of gene expression levels between the ceased period and laying period libraries. DEGs were filtered using FDR ≤0.001 and |log_2_ratio| ≧1 as a threshold. Red spots represent up-regulated genes, and green spots indicate down-regulated genes. Blue spots represents genes that did not show obvious changes between the ceased period and laying period samples.

To discover the differentially expressed genes between the ceased period and laying period, the gene expression levels were calculated using the RPKM method (Reads per Kb per Million mapped reads). Between the two libraries, 12120 entities were detected, and according to the applied criteria (FDR ≤0.001 and |log_2_ratio |≧1), 688 significantly differentially expressed genes composed of 344 up-regulated and 344 down-regulated genes were identified (Table S2, Figure 5), which included genes related to reproduction and reproductive processes, sex steroid hormone biosynthesis, and hormone secretion, such as D(2) dopamine receptor (DRD2), progesterone receptor (PGR), lutropin-choriogonadotropic hormone receptor precursor (LHCGR), insulin receptor isoform 1 (INSR), prolactin receptor precursor (PRLR), secretogranin-2 (SCG2), steroid hormone receptor ERR2 (ESRRB), melatonin receptor type 1C (MEL1C), neuropeptide Y receptor type 1 (NPY1R), growth hormone receptor (GHR), vasoactive intestinal polypeptide receptor 2 (VIPR2), steroidogenic acute regulatory protein (STAR), 3 beta-hydroxysteroid dehydrogenase 1 (HSD3B2), cholesterol side-chain cleavage enzyme (CYP11A1), steroid 17-alpha-hydroxylase/17, 20 lyase (CYP17A1), cytochrome P450 19A1 (CYP19A1), and testosterone 17-beta-dehydrogenase 3 (HSD17B3).

### Functional annotation of differentially expressed genes

To determine the function of differentially expressed genes, all of the DEGs in this study were mapped to terms in the GO database. A total of 1431 genes were categorized into the three main categories of GO classification, with 33.8% (484) categorized as cellular components, 32.3% (462) categorized as molecular functions, and 33.9% (485) categorized as biological processes (Table S3). For cellular components, genes involved in the cell (GO: 0005623, 392, 81%) and cell part (GO: 0044464, 392, 81%) were highly represented and followed by the intracellular (GO: 0005622, 359, 74.2%) and intracellular part (GO: 0044424, 359, 74.2%). For molecular functions, binding (GO: 0005488, 383, 82.9%) was the most representative GO term, followed by catalytic activity (GO: 0003824, 181, 39.2%) and heterocyclic compound binding (GO: 1901363, 142, 30.7%). The most well-represented of the biological processes category was cellular processes (GO: 0009987, 419, 86.4%), followed by single-organism processes with 370 genes (GO: 0044699, 76.3%) and single-organism cellular processes (GO: 0044763, 331, 68.2%). As expected, certain genes were involved in “reproduction” and “reproductive processes” (Table S4).

A KEGG pathway analysis allowed us to determine functional classes of the differentially expressed genes during different egg-laying stages. In this study, a total of 585 genes mapped onto 214 pathways were selected following this process. A summary of the genes involved in these pathways has been included in Table S5. The largest category was metabolic pathways (Ko01100, 12.14%), which had 71 annotated genes. Notably, a specific enrichment of genes was observed for pathways involved in reproduction regulation, such as steroid hormone biosynthesis, GnRH signaling pathways, oocyte meiosis, progesterone-mediated oocyte maturation, steroid biosynthesis, calcium signaling pathways, G-protein coupled receptor signaling pathway, dopaminergic synapse, and MAPK signaling pathway (Table 2). Specifically, several genes are involved in multiple pathways, such as ADCY7 and ADCY2 (progesterone-mediated oocyte maturation, GnRH signaling pathways, oocyte meiosis, calcium signaling pathways, G-protein coupled receptor signaling pathway), PRKX (progesterone-mediated oocyte maturation, GnRH signaling pathways, oocyte meiosis, calcium signaling pathways, dopaminergic synapse, MAPK signaling pathway), PRKCB (GnRH signaling pathways, calcium signaling pathways, dopaminergic synapse, MAPK signaling pathway), PGR (progesterone-mediated oocyte maturation, oocyte meiosis), and LHCGR (calcium signaling pathways, G-protein coupled receptor signaling pathway).

**Table 2:** Putative candidate genes of the DEGs associated with goose laying.

Gene	Gene ID	log2 Ratio(laying_period/ceased_period)	Up-Down-Regulation(laying_period/ceased_period)
**Steroid hormone biosynthesis**			
cytochrome P450 3A40 (CYP3A4)	gi|513207965|	1.158476902	Up
testosterone 17-beta-dehydrogenase 3 (ZNF367)	gi|513231050|	−5.619898953	Down
beta-hydroxysteroid dehydrogenase 1 (HSD3B2)	gi|45384115|	−5.261921985	Down
cholesterol side-chain cleavage enzyme, mitochondrial (CYP11A1)	gi|48976108|	−3.359829329	Down
steroid 17-alpha-hydroxylase/17,20 lyase precursor (CYP17A1)	gi|402692072|	−3.202412536	Down
estradiol 17-beta-dehydrogenase 1 (HSD17B1)	gi|45382290|	−1.429215392	Down
cytochrome P450 19A1 (CYP19A1)	gi|48976118|	−1.248643146	Down
**Progesterone-mediated oocyte maturation**			
progesterone receptor (PGR)	gi|45383981|	1.432343028	Up
phosphoinositide 3-kinase regulatory subunit 5 (PIK3R5)	gi|71897362|	1.408455155	Up
adenylate cyclase type 7 (ADCY7)	gi|513203244|	1.319161648	Up
fizzy-related protein homolog isoform 1 (CDH1-A)	gi|493795026|	1.278954323	Up
serine/threonine-protein kinase PLK1 (PLK1)	gi|71894744|	1.011346258	Up
uncharacterized protein LOC416455 (C7ORF50)	gi|513207883|	−1.534242869	Down
LOW QUALITY PROTEIN: adenylate cyclase type 2 (ADCY2)	gi|513171076|	−1.518812828	Down
heat shock protein HSP 90-alpha (HSP90AA1)	gi|157954046|	−1.353494256	Down
cAMP-dependent protein kinase catalytic subunit PRKX (PRKX)	gi|513164084|	−1.249276602	Down
N-chimaerin (CHN1)	gi|61098445|	−1.162811275	Down
**GnRH signaling pathway**			
cytosolic phospholipase A2 epsilon-like	gi|513188970|	2.695250609	Up
protein kinase C beta type (PRKCB)	gi|513209045|	1.328811818	Up
adenylate cyclase type 7 (ADCY7)	gi|513203244|	1.319161648	Up
dual specificity mitogen-activated protein kinase kinase 3 (MAP2K3)	gi|497815303|	1.306754897	Up
voltage-dependent L-type calcium channel subunit alpha-1D (CACNA1D)	gi|402232741|	−1.676888932	Down
LOW QUALITY PROTEIN: adenylate cyclase type 2 (ADCY2)	gi|513171076|	−1.518812828	Down
cAMP-dependent protein kinase catalytic subunit PRKX (PRKX)	gi|513164084|	−1.249276602	Down
copine-8 (CPNE8)	gi|313747427|	−1.166180986	Down
**Oocyte meiosis**			
progesterone receptor (PGR)	gi|45383981|	1.432343028	Up
adenylate cyclase type 7 (ADCY7)	gi|513203244|	1.319161648	Up
serine/threonine-protein kinase PLK1 (PLK1)	gi|71894744|	1.011346258	Up
structural maintenance of chromosomes protein 1B (SMC1B)	gi|513160707|	−1.624471683	Down
LOW QUALITY PROTEIN: adenylate cyclase type 2 (ADCY2)	gi|513171076|	−1.518812828	Down
cAMP-dependent protein kinase catalytic subunit PRKX (PRKX)	gi|513164084|	−1.249276602	Down
**Steroid biosynthesis**			
7-dehydrocholesterol reductase (DHCR7)	gi|313747478|	−2.132003871	Down
24-dehydrocholesterol reductase (DHCR24)	gi|71896814|	−1.780922052	Down
methylsterol monooxygenase 1 (MSMO1)	gi|57530154|	−1.744980748	Down
**Calcium signaling pathway**			
glutamate [NMDA] receptor subunit epsilon-3-like (GRIN2C)	gi|513214949|	3.069035476	Up
platelet-derived growth factor receptor beta (PDGFRB)	gi|513207013|	2.483913376	Up
protein AHNAK2-like	gi|513190197|	2.262291202	Up
protein kinase C beta type (PRKCB)	gi|513209045|	1.328811818	Up
adenylate cyclase type 7 (ADCY7)	gi|513203244|	1.319161648	Up
voltage-dependent T-type calcium channel subunit alpha-1I (CACNA1I)	gi|513159170|	1.316738986	Up
inositol-trisphosphate 3-kinase B (ITPKB)	gi|513174490|	1.297879958	Up
voltage-dependent T-type calcium channel subunit alpha-1H (CACNA1H)	gi|513208286|	1.161115576	Up
voltage-dependent N-type calcium channel subunit alpha-1B (CACNA1B)	gi|45383553|	1.100472667	Up
lutropin-choriogonadotropic hormone receptor precursor (LHCGR)	gi|45384387|	−2.357780254	Down
type-1 angiotensin II receptor (AGTR1)	gi|119943121|	−1.853766694	Down
voltage-dependent L-type calcium channel subunit alpha-1D (CACNA1D)	gi|402232741|	−1.676888932	Down
LOW QUALITY PROTEIN: adenylate cyclase type 2 (ADCY2)	gi|513171076|	−1.518812828	Down
1-phosphatidylinositol 4,5-bisphosphate phosphodiesterase epsilon-1	gi|513191366|	−1.380738319	Down
cAMP-dependent protein kinase catalytic subunit PRKX (PRKX)	gi|513164084|	−1.249276602	Down
copine-8 (CPNE8)	gi|313747427|	−1.166180986	Down
**G-protein coupled receptor signaling pathway**			
neuropeptide Y receptor type 1 (NPY1R)	gi|71895854|	1.234524222	Up
probable G-protein coupled receptor 133 (GPR133)	gi|513209875|	2.254811236	Up
diacylglycerol kinase zeta (DGKZ)	gi|71894854|	1.067874353	Up
low-density lipoprotein receptor-related protein 1 precursor (LRP1)	gi|45382556|	1.41426879	Up
Agrin C-terminal 22 kDa fragment (AGRN)	gi|45382976|	1.005732177	Up
diacylglycerol kinase beta-like (DGKG)	gi|513199158|	1.178410829	Up
filamin-A (FLNA)	gi|45383034|	1.326016799	Up
adenylate cyclase type 7 (ADCY7)	gi|513203244|	1.319161648	Up
alpha-2A adrenergic receptor (ADRA2A)	gi|513191949|	2.134988549	Up
VPS10 domain-containing receptor SorCS2 (SORCS2)	gi|513184939|	1.409219902	Up
D(2) dopamine receptor (DRD2)	gi|164518969|	1.792318027	Up
gamma-aminobutyric acid receptor subunit alpha-1 precursor (GABRA1)	gi|319655698|	−1.42313234	Down
neuron-specific protein family member 2-like isoform 1	gi|513206640|	−2.356864548	Down
gamma-aminobutyric acid receptor subunit gamma-4 precursor (GABRE)	gi|46048734|	−1.882084965	Down
lutropin-choriogonadotropic hormone receptor precursor (LHCGR)	gi|45384387|	−2.357780254	Down
type-1 angiotensin II receptor (AGTR1)	gi|119943121|	−1.853766694	Down
G protein-coupled receptor kinase 5 (GRK5)	gi|513192176|	−1.585133535	Down
LOW QUALITY PROTEIN: diacylglycerol kinase theta (DGKQ)	gi|513232846|	−1.013456725	Down
voltage-dependent L-type calcium channel subunit alpha-1D (CACNA1D)	gi|402232741|	−1.676888932	Down
1-phosphatidylinositol 4,5-bisphosphate phosphodiesterase epsilon-1	gi|513191366|	−1.380738319	Down
mu-type opioid receptor-like, partial (OPRM1)	gi|513176530|	−8.310406357	Down
LOW QUALITY PROTEIN: adenylate cyclase type 2 (ADCY2)	gi|513171076|	−1.518812828	Down
**dopaminergic synapse**			
D(2) dopamine receptor (DRD2)	gi|164518969|	1.792318027	Up
protein kinase C beta type (PRKCB)	gi|513209045|	1.328811818	Up
voltage-dependent N-type calcium channel subunit alpha-1B (CACNA1B)	gi|45383553|	1.100472667	Up
guanine nucleotide-binding protein G(I)/G(S)/G(O) subunit gamma-11 (GNG11)	gi|487442755|	−1.075578437	Down
neuron-specific protein family member 2-like isoform 1	gi|513206640|	−2.356864548	Down
voltage-dependent L-type calcium channel subunit alpha-1D (CACNA1D)	gi|402232741|	−1.676888932	Down
uncharacterized protein LOC416455	gi|513207883|	−1.534242869	Down
cAMP-dependent protein kinase catalytic subunit PRKX (PRKX)	gi|513164084|	−1.249276602	Down
copine-8 (CPNE8)	gi|313747427|	−1.166180986	Down
N-chimaerin (CHN1)	gi|61098445|	−1.162811275	Down
**MAPK signaling pathway**			
cytosolic phospholipase A2 epsilon-like	gi|513188970|	2.695250609	Up
platelet-derived growth factor receptor beta (PDGFRB)	gi|513207013|	2.483913376	Up
dual specificity protein phosphatase 4 (DUSP4)	gi|45382296|	2.176808724	Up
protein kinase C beta type (PRKCB)	gi|513209045|	1.328811818	Up
filamin-A (FLNA)	gi|45383034|	1.326016799	Up
voltage-dependent T-type calcium channel subunit alpha-1I (CACNA1I)	gi|513159170|	1.316738986	Up
TGF-beta receptor type-2 precursor (TGFBR2)	gi|45382188|	1.310075255	Up
dual specificity mitogen-activated protein kinase kinase 3 (MAP2K3)	gi|497815303|	1.306754897	Up
filamin-B (FLNB)	gi|71896430|	1.217226477	Up
voltage-dependent T-type calcium channel subunit alpha-1H (CACNA1H)	gi|513208286|	1.161115576	Up
voltage-dependent N-type calcium channel subunit alpha-1B (CACNA1B)	gi|45383553|	1.100472667	Up
serum response factor (SRF)	gi|356460963|	1.090757769	Up
nuclear factor NF-kappa-B p105 subunit (NFKB1)	gi|45384085|	1.084126722	Up
stathmin-2 (STMN2)	gi|49170101|	−3.928021249	Down
keratinocyte growth factor (FGF7)	gi|60302701|	−2.27197565	Down
fibroblast growth factor 12 (FGF12)	gi|45382434|	−2.240405571	Down
voltage-dependent L-type calcium channel subunit alpha-1D (CACNA1D)	gi|402232741|	−1.676888932	Down
platelet-derived growth factor subunit B precursor (PDGFB)	gi|326693934|	−1.449973952	Down
cAMP-dependent protein kinase catalytic subunit PRKX (PRKX)	gi|513164084|	−1.249276602	Down
tyrosine-protein phosphatase non-receptor type 5 (PTPN5)	gi|513186533|	−1.227581531	Down
microtubule-associated protein tau (MAPT)	gi|312836786|	−1.185761355	Down
copine-8 (CPNE8)	gi|313747427|	−1.166180986	Down
N-chimaerin (CHN1)	gi|61098445|	−1.162811275	Down
neuron navigator 3 (NAV3)	gi|433660829|	−1.050703769	Down

### Quantitative real-time PCR validation

**Figure 6:**
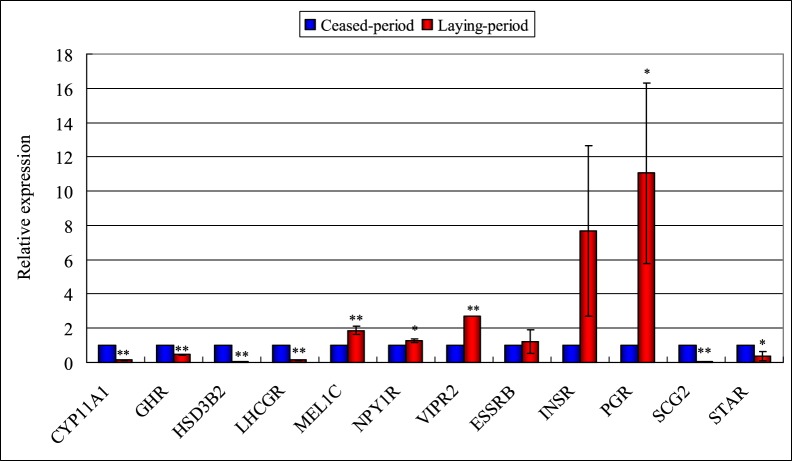
Validation of the gene expression profile by qRT-PCR. Total RNA extracted from the ovarian tissues that was measured by RT-PCR analysis; relative expression levels were calculated according to the 2^−ΔΔCT^ method using beta-actin as an internal reference gene and the ceased period group as the calibrator (relative expression  = 1). Error bar shows the standard deviation. Gene symbols indicating different genes refer to Table 2. *P<0.05, **P<0.01.

As shown in Figure 6 and Table 3, the general expression patterns of twelve genes from the Illumina sequencing are consistent with the qRT-PCR results, which further support the reliability of the Illumina sequencing data. The discrepancies with respect to ratio may be attributed to the essentially different algorithms and sensitivities between the two techniques.

**Table 3:** Evaluation of the expression profile variation between RNA-Seq and qRT-PCR for selected genes.

Gene	Gene ID	Description	Fold change (laying_period/ceased_period)		*P* value	
			RNA-Seq	qRT-PCR	RNA-Seq	qRT-PCR
PGR	gi|45383981|	progesterone receptor [Gallus gallus]	1.43	11.04	6.05E-11	3.0000E-02
INSR	gi|513227719|	PREDICTED: insulin receptor isoform 1 [Gallus gallus]	1.70	7.69	4.73E-17	1.4540E-01
NPY1R	gi|71895854|	neuropeptide Y receptor type 1 [Gallus gallus]	1.24	1.27	6.90E-08	3.60E-02
ESRRB	gi|513188405|	PREDICTED: steroid hormone receptor ERR2 [Gallus gallus]	1.03	1.21	0.000128778	6.4259E-01
MEL1C	gi|45382488|	melatonin receptor type 1C [Gallus gallus]	1.74	1.86	4.76E-05	3.00E-03
VIPR2	gi|62461589|	vasoactive intestinal polypeptide receptor 2 precursor [Gallus gallus]	1.09	2.69	5.30E-09	0
LHCGR	gi|45384387|	lutropin-choriogonadotropic hormone receptor precursor [Gallus gallus]	−2.36	0.14	1.15E-29	0
SCG2	gi|513199280|	PREDICTED: secretogranin-2 [Gallus gallus]	−2.26	0.05	6.99E-38	0
GHR	gi|47604939|	growth hormone receptor precursor [Gallus gallus]	−1.51	0.46	2.39E-10	0
STAR	gi|402692505|	steroidogenic acute regulatory protein, mitochondrial precursor [Gallus gallus]	−3.11	0.36	1.46E-92	4.9000E-02
HSD3B2	gi|45384115|	3 beta-hydroxysteroid dehydrogenase 1 [Gallus gallus]	−5.26	0.03	0	0
CYP11A1	gi|48976108|	cholesterol side-chain cleavage enzyme, mitochondrial [Gallus gallus]	−3.36	0.14	1.99E-229	0

## Discussion

During formation of the egg in the bird egg-laying cycle, the ovary undergoes dynamic hormonal, biochemical and cellular changes. The dynamic and highly regulated process of follicle development requires the coordinated actions of a great number of genes orchestrated at the transcriptional and posttranscriptional levels [24]. A number of researchers have proven that transcriptome sequencing of RNA provides a rapid and cost-effective method of generating whole-transcriptome sequences from numerous types of biological samples and identifying differentially expressed genes. In the present study, the Illumina sequencing method was used to analyze the transcriptome of goose ovarian tissue at two stages of the egg-laying cycle and identified 344 up-regulated and 344 down-regulated genes in the laying period compared with ceased period. Some of those involved in steroid hormone biosynthesis, GnRH signaling pathways, oocyte meiosis, progesterone-mediated oocyte maturation, dopaminergic synapse, steroid biosynthesis, calcium signaling pathways, G-protein coupled receptor signaling pathway, and MAPK signaling pathway.

Poultry reproduction is tightly regulated by sex steroid hormones. Sex steroid hormones, such as progesterone and estradiol, are involved in the regulation of ovulation, gonadal differentiation, and sexual and nesting behaviors in birds through interactions with their intracellular receptors [25]. The hormone progesterone exerts a regulatory function on follicular development. The follicular and ovulation stages are dependent on gonadotropin release. In poultry production, progesterone is secreted by follicle granulose cells, and the target sites of progesterone action are the oviduct and hypothalamic-pituitary-ovarian axis. The secretion of progesterone is tightly correlated with the growth and status of the ovary. In the ovulation process, progesterone acts as a positive feedback signal to trigger the GnRH/LH pulse surge prior to ovulation [26]. Similarly to all steroid hormones, progesterone induces its biological effects by binding to an intracellular protein, which is the progesterone receptor (PGR) in this case. The PGR is localized in target tissues such as the brain, ovary and oviduct[27], [28], [29] , and its expression is regulated by several hormones, such as estradiol, progesterone and gonadotropins [30]. The expression of PGR is up-regulated after FSH treatment and down-regulated after LH treatment in the interstitial cells of chick ovarian medulla [31]. In the ovaries of laying hens after estrogen treatment, there is an increased number of PGR-positive stromal, thecal and granulosa cells [32]. Furthermore, a significant decrease in PGR concentration is observed in all of the cell types of chick oviducts after injection of progesterone [33]. In the present study, the expression of PGR is up-regulated in the laying period, which is consistent with progesterone acting via its receptor to affect the regulation of follicular maturation, ovulation and oviposition in poultry.

Prolactin (PRL) is a single-chain peptide hormone that is primarily synthesized by the anterior pituitary and belongs to the prolactin/growth hormone (GH) family [34]. In birds, PRL has been reported to be involved in the regulation of many important physiological processes, including egg laying, induction and maintenance of incubation behavior, osmoregulation, immune-modulation, and gonadal development and functions [35], [36]. Notably, PRL plays an inhibitory role in avian reproductive activities at all levels of the hypothalamic-pituitary-gonadal axis via inhibition of gonadotropin secretion, stimulation of incubation behavior, and development of atresia in ovarian follicles [37], [38]. The role of PRL is to specifically combine with prolactin receptor (PRLR) in the membrane of effector cells. PRLR belongs to the class I cytokine receptor superfamily [39]. In adult chickens, turkeys and geese, the PRLR is widely distributed in various tissues [40], [41], and several studies have indicated that PRLR may play an important role in ovarian development and the egg-laying process in geese [39], [42], [43]. The highest level of expression of PRLR in the ovaries of Eastern Zhejiang White Geese was found at the incubation stage; a lower level of expression occurred during the egg-laying stage, and the lowest level of expression was found in the out-of-lay stage [44]. Our transcriptome analysis showed that the expression of PRLR in the laying period was significantly decreased compared to that of the ceased period. Therefore, the down-regulation of PRLR might be reducing the inhibitory effect of PRL on reproductive activities.

The production of steroid hormones by ovarian cells is essential for follicular recruitment, oocyte maturation, ovulation, and hypothalamo-pituitary-gonadal (HPG) axis regulation. Steroidogenesis is therefore integral to successful reproduction. Sex steroid hormones are derived from cholesterol through a series of enzyme-catalyzed reactions. First, cholesterol is transported from the outer to the inner mitochondrial membrane by the steroid acute regulatory protein (STAR) [45], [46], [47]. At the cytochrome P450 cholesterol side-chain cleavage (P450scc) enzyme site (encoded by CYP11A1), CYP11A1 cleaves the side chain of cholesterol and converts it to pregnenolone [48]. Pregnenolone is further catalyzed by other steroidogenic enzymes, which HSD3B2 catalyze the conversion of pregnenolone to progesterone, 17-hydroxypregnenolone to 17-hydroxyprogesterone (17OHP), dehydroepiandrosterone (DHEA) to androstenedione and androstenediol to testosterone. CYP17A1 is responsible for the conversion of pregnenolone to 17-hydroxypregnenolone, progesterone to 17-hydroxyprogesterone (17OHP), 17OHP to androstenedione and 17-hydroxypregnenolone to DHEA. HSD17B1 catalyzes the conversion of DHEA to androstenedione and estrone to estradiol, HSD17B3 catalyzes the conversion of androstenedione into testosterone, and CYP19A1 catalyzes the conversion of testosterone to estradiol and androstenedione to estrone [49]. Among these enzymes, STAR and CYP11A1 are the first and rate-limiting steps in the steroidogenic pathway. In avian species, the ovary is organized into a stroma with primordial follicles (<1 mm in diameter), small white follicles (1 to 4 mm), yellowish follicles (4 to 8 mm), and a hierarchy of yellow follicles (F_n_ to F_1_; 9 to 35 mm) that ovulate on successive days. The process of growth and maturation of the ovarian follicle is associated with the differentiation of granulosa cells, which occurs before selection of the yellowish follicle into the preovulatory hierarchy [50], [51]. During the transition of the prehierarchical follicles to a preovulatory hierarchy, the cells of the granulosa layer, which were initially stimulated by FSH and subsequently by LH, begin to express the STAR protein and CYP11A1 and produce progesterone (P_4_), which is catalyzed by HSD3Bs [46], [52]. Numerous investigations have suggested that the elevated P_4_ production in the granulosa layers of the F_3_→F_1_ follicles is predominantly associated with an increasing expression of STAR, CYP11A1 and HSD3B mRNAs [26], [47], [53], [54] and enhanced HSD3B activity [55]. CYP17A1, which has both 17 alpha-hydroxylase and 17, 20-lyase activities, is a key regulatory enzyme in the steroidogenic pathway. In the gonads, CYP17 deficiency prevents gonadal sex steroid production and leads to female external genitalia development [56]. CYP19A1 and HSD17B1 are responsible for estrogen biosynthesis. After prenatal and neonatal flutamide exposure in porcine ovarian follicles, the elevated level of 17-estradiol is associated with overexpression of the follicle stimulating hormone receptor (FSHR), CYP19A1, and CTNNB1 genes [57]. However, despite the role of the key enzymes of the steroid synthesis pathway, steroid hormones have a negative feedback effect on steroidogenic enzyme expression and regulate their own production in ovarian tissue [58], [59]. Notably, the genes encoding steroidogenic enzymes in our study were down-regulated in the laying period compared with the ceased period, which may be related to the negative feedback regulation of sex hormones on steroidogenic enzyme expression during the egg-laying period.

In addition to the above-mentioned genes, several G-protein-coupled receptors, including DRD2, VIPR2, NPY1R, and MEL1C, were identified. These proteins play key roles in the regulation of steroid hormone biosynthesis and reproductive functions. Dopamine belongs to a group of neurotransmitters called catecholamines and exerts its action by binding to specific membrane receptors. Dopamine receptors are members of the G-protein-coupled receptors with seven transmembrane domains [60]. In avian species, two distinct dopamine receptor subtypes, DRD1 and DRD2, have been reported. Dopamine can inhibit PRL secretion via DRD2 at the pituitary level by operating through vasoactive intestinal peptide (VIP) [61]. The DRD2 gene is widely distributed in the hypothalamic-pituitary-ovarian axis of chickens, turkeys and geese, and it plays an important role in the regulation of hormone secretion and reproductive behaviors [62], [63], [64]. The elevation of DRD2 expression during the Huoyan goose egg-laying period might be capable of down-regulating the expression of PRL. VIP belongs to a family of regulatory peptides, and in birds, a major role of VIP involves regulating the synthesis and secretion of the anterior pituitary hormone prolactin (PRL). VIP can increase the transcription of the gene encoding PRL, enhance PRL mRNA stability, and elevate hormone secretion [65]. The actions of VIP are mediated through an interaction with two receptor types (VIPR1 and VIPR2) that belong to the class II subfamily of the 7-transmembrane G-protein-coupled receptor superfamily. VIP receptors are present on the surface membranes of the anterior pituitary, hypothalamus, small intestine and granulosa cells [66]. The VIPR1 gene in the pituitary is involved in the regulation of reproductive activity in avian species [67]. The expression of VIP receptor mRNA in the pituitary is markedly reduced in nonphotostimulated and photorefractory turkey hens compared with laying and incubating hens [68]. In the ovaries of Huoyan geese during the egg-laying period (April–August), the rise of VIPR2 expression might be a result of the long photoperiod. Neuropeptide Y (NPY) is known to participate in the regulation of several physiological functions, such as gonadotropin release, sexual behavior, food intake, energy metabolism, and stress responses. In birds, NPY has multiple functions related to the HPG system and GnRH secretion [69], [70]. The great variety of physiological functions of NPY is mediated through G-protein-coupled receptors (NPY1R, NPY5R) [71], [72], [73]. Melatonin plays an important role in the regulation of animal reproduction via binding to G-protein-coupled receptors. Three melatonin receptor subtypes (Mel-1a, Mel-1b and Mel-1c) have been shown to be differentially expressed in chicken ovarian granulosa cells [74]. In the gonads of birds, melatonin and its receptors play an important role in regulating the viability and maturation of ovarian granular cells, gonadal steroidogenesis, folliculogenesis and maturation of oocytes [75]. Changes in the expression levels of the melatonin receptors and estrogen concentrations in the hierarchical follicles of the Sichuan white goose are consistent with the changes that occur in the Huoyan goose [76]. In our study, the increase of Mel-1C expression during the egg-laying period suggests that melatonin may regulate ovarian function through its receptor.

In both avian and mammalian species, the process of ovarian follicle maturation is coupled with a functional differentiation of the granulosa cell layer. granulosa cells from mature, preovulatory follicles and granulosa-luteal cells produce appreciable amounts of steroid hormones [77]. granulosa cell differentiation and steroidogenesis during follicle maturation are mediated by the coordinately timed expression of endocrine, paracrine, and autocrine factors and the interaction of consequent signaling pathways [78]. For instance, in ovarian steroidogenic cells, gonadotropins bind to specific membrane G-protein coupled receptors (GPCRs) and lead to the activation of multiple signal transduction pathways, including the adenylate cyclase-/cAMP-dependent protein kinase A (PKA) signaling pathway and calcium-/calmodulindependent pathways [79]. Studies indicate that activation of the mitogen-activated protein kinase (MAPK) and protein kinase C (PKC) signaling pathways modulates steroid biosynthesis during follicle development [80]. In our study, list of differentially expressed genes are involved in pathways calcium signaling pathways, G-protein coupled receptor signaling pathway, and MAPK signaling pathway. Like PRKCB, ADCY7, PRKX, MAP2K3, and TGFBR2, their roles in follicle development and productivity of goose need to be further investigated.

## Conclusions

In conclusion, this study presents the transcriptomic profiles of ovarian tissue from Huoyan geese during the ceased and laying periods using Illumina RNA-Seq technology. Differentially expressed genes which involved in pathways for reproduction regulation, such as steroid hormone biosynthesis, GnRH signaling pathways, oocyte meiosis, progesterone-mediated oocyte maturation, steroid biosynthesis, calcium signaling pathways, G-protein coupled receptor signaling pathway, dopaminergic synapse, and MAPK signaling pathway were identified, furthermore, the expression profiles were validated by qRT-PCR. These data provide comprehensive gene expression information at the transcriptional level that might increase our understanding of the Huoyan goose's reproductive biology.

## Supporting Information

Table S1 **Primers used for the quantitative real-time PCR analysis.**
(DOC)

Table S2 **Differentially expressed genes between the ceased period and laying period ovaries.**
(XLS)

Table S3 **GO classification of differentially expressed genes.** A list of GO terms enriched for differentially expressed genes showing higher transcript abundance in goose ovaries.
(XLS)

Table S4 **Differentially expressed genes involved in reproduction and reproductive process.**
(DOC)

Table S5 **KEGG pathway annotation of differentially expressed genes.** A list of KEGG pathways enriched for differentially expressed genes showing higher transcript abundance in goose ovaries.
(XLS)
