# Supplementary material for: Transcriptome Profiling Identifies Differentially Expressed Genes in Huoyan Goose Ovaries between the Laying Period and Ceased Period
Source: PLoS One. 2014 Nov 24;9(11):e113211. doi: 10.1371/journal.pone.0113211 (PMC4242529; doi:10.1371/journal.pone.0113211)
Supplement: Table S1 — Primers used for the quantitative real-time PCR analysis. (DOC) [file pone.0113211.s001.doc]

**Table S1 Primers used for the quantitative real-time RT-PCR analysis**.

| Gene Symbol | Gene ID | Forward primer (5′-3′) | Reverse primer (5′-3′) | Product length(bp) |
| --- | --- | --- | --- | --- |
| INSR | gi|513227719| | TGAGACCCGACGCTGAGAATA | GATATCTCTGGTCATGCCGAAGT | 200 |
| SCG2 | gi|513199280| | CAATACGAACCAGATGAAACGAGTT | CCTCGTCCAGATACTGCCTGTT | 200 |
| NPY1R | gi|71895854| | TTCGGAGAAGAACTCGCAGA | TATCACAGTCCCGTAAGCCAAG | 142 |
| PGR | gi|45383981| | CCAGAAAGGTGTGGTGGCTAAC | TCACCATCCCTGCCAGAATC | 200 |
| ESRRB | gi|513188405| | GAAGGTGTTCGTCTGGATCGA | ATGTCACTCTCCGGCATGGT | 200 |
| LHCGR | gi|45384387| | AGCTGTGTGACAACTTGCGTATGAC | CATTGAAGGCATGGCTGTGG | 126 |
| MEL1C | gi|45382488| | TGAGACAAGACTGCAAGCAGAAG | CCCCAGCACACAGCAAAA | 94 |
| GHR | gi|47604939| | GAGGTATGGATCTTCGGCATCTG | GTGACCTGCACTTGCTGATTTG | 114 |
| HSD17B1 | gi|45382290| | TGAGAGCAGTGTTTGAGGTGAAC | CGCAGTACACGGCGTTGA | 150 |
| STAR | gi|402692505| | AGGAAGCCCTGCAGAAATCA | GTCGCCCATCTGCTCCAT | 200 |
| HSD3B2 | gi|45384115| | AAGATGAGGCGCTGGCTGA | CAATGATGGAAGCTGTGTGGATG | 186 |
| CYP11A1 | gi|48976108| | ATACCGTGACTACCGCAACAAG | TCCAGCAAGGGCACGAA | 132 |
| VIPR2 | gi|62461589| | TGGTGTCCATCCAGAATGCAG | AGGTGATGTTATCCCAAACTCCAA | 128 |
| CYP19A1 | gi|48976118| | TTGCATCTCAGCTGATTTTTGC | CTCTCTGTCACCCATAACAGTTTCA | 200 |
| ACTB | gi|45382926| | ATTGTCCACCGCAAATGCTTC | AAATAAAGCCATGCCAATCTCGTC | 113 |
