# Supplementary material for: Transcriptome Profiling Identifies Differentially Expressed Genes in Huoyan Goose Ovaries between the Laying Period and Ceased Period
Source: PLoS One. 2014 Nov 24;9(11):e113211. doi: 10.1371/journal.pone.0113211 (PMC4242529; doi:10.1371/journal.pone.0113211)
Supplement: Table S4 — Differentially expressed genes involved in reproduction and reproductive process. (DOC) [file pone.0113211.s004.doc]

Table S4. Differentially expressed genes involved in reproduction and reproductive process.

| Gene | GeneID | log2 Ratio  (laying_period/ceased_period) | Up-Down-Regulation  (laying_period/ceased_period) |
| --- | --- | --- | --- |
| rhomboid domain-containing protein 1 (RHBDD1) | gi|513199307| | 2.187455969 | Up |
| keratin, type I cytoskeletal 19 (KRT19) | gi|485049500| | 1.816812589 | Up |
| D(2) dopamine receptor (DRD2) | gi|164518969| | 1.792318027 | Up |
| protein C-ets-1 (ETS1) | gi|212720680| | 1.786224269 | Up |
| POU domain, class 2, transcription factor 3 (POU2F3) | gi|513222360| | 1.74204482 | Up |
| insulin receptor isoform 1 (INSR) | gi|513227719| | 1.702709497 | Up |
| gap junction beta-2 protein (GJB2) | gi|399154153| | 1.630399464 | Up |
| tyrosine-protein kinase Mer precursor (MERTK) | gi|45384491| | 1.627683376 | Up |
| zona pellucida sperm-binding protein 2 precursor (ZP2) | gi|84993246| | 1.588335405 | Up |
| tenascin precursor (TNC) | gi|312032349| | 1.522440216 | Up |
| progesterone receptor (PGR) | gi|45383981| | 1.432343028 | Up |
| zona pellucida sperm-binding protein 4 (ZP4) | gi|45382406| | 1.429530756 | Up |
| zinc finger and BTB domain-containing protein 16 (ZBTB16) | gi|513222307| | 1.383853181 | Up |
| B-cell lymphoma 6 protein homolog (BCL6) | gi|61098401| | 1.283851935 | Up |
| Myotubularin-related protein 5 (SBF1) | gi|513157596| | 1.279625813 | Up |
| fizzy-related protein homolog isoform 1 (CDH1-A) | gi|493795026| | 1.278954323 | Up |
| protein tyrosine phosphatase, non-receptor type 11-like | gi|357593600| | 1.27491881 | Up |
| integrin beta-3 precursor (ITGB3) | gi|46048953| | 1.257845296 | Up |
| bone morphogenetic protein 7, partial (BMP7) | gi|513219057| | 1.198344285 | Up |
| cystathionine beta-synthase (CBS) | gi|513163069| | 1.187455969 | Up |
| mothers against decapentaplegic homolog 3 (SMAD3) | gi|45383212| | 1.144836335 | Up |
| homeobox protein Lhx8 (LHX8) | gi|402743403| | 1.120637862 | Up |
| beta-1,3-N-acetylglucosaminyltransferase lunatic fringe precursor (LFNG) | gi|45384413| | 1.108021501 | Up |
| LOW QUALITY PROTEIN: pappalysin-1 (PAPPA) | gi|513212106| | 1.102123915 | Up |
| signal transducer and activator of transcription 5B (STAT5B) | gi|45382144| | 1.044498015 | Up |
| EF-hand domain-containing protein C3orf25 homolog (RPL32) | gi|356582406| | -1.012100364 | Down |
| protein DJ-1 (PARK7) | gi|45383014| | -1.016077425 | Down |
| ribosomal protein S3A (RPS3A) | gi|160333402| | -1.033380599 | Down |
| 60S ribosomal protein L36 (RPL36) | gi|45383831| | -1.039136441 | Down |
| cartilage-associated protein precursor (CRTAP) | gi|45384145| | -1.053008026 | Down |
| 60S ribosomal protein L18a, partial (RPL18A) | gi|513158353| | -1.070040254 | Down |
| 60S ribosomal protein L11 (RPL11) | gi|71896866| | -1.090633852 | Down |
| thymidylate synthase (TYMS) | gi|513172723| | -1.096877839 | Down |
| 40S ribosomal protein S8 (RPS8) | gi|356460916| | -1.109827999 | Down |
| mCG7602 (RPS29) | gi|482677671| | -1.116264508 | Down |
| Phosphatidylinositol-4-phosphate 3-kinase C2 domain-containing subunit alpha (RPS13) | gi|49169797| | -1.127214572 | Down |
| 60S ribosomal protein L5 (RPL5) | gi|148762941| | -1.157131029 | Down |
| 40S ribosomal protein S14-like (RPS14) | gi|71895290| | -1.157143789 | Down |
| 60S ribosomal protein L27-like (RPL27) | gi|45384445| | -1.17667336 | Down |
| arylsulfatase A (ARSA) | gi|513157191| | -1.204861454 | Down |
| stem cell growth factor receptor Kit precursor (KIT) | gi|45383437| | -1.236919395 | Down |
| 60S ribosomal protein L31 (RPL31) | gi|480306425| | -1.241104975 | Down |
| cytochrome P450 19A1 (CYP19A1) | gi|48976118| | -1.248643146 | Down |
| 60S ribosomal protein L9 (RPL9) | gi|382545824| | -1.257232337 | Down |
| angiopoietin-2 (ANGPT2) | gi|46048869| | -1.268453812 | Down |
| 40S ribosomal protein S15 (RPS15) | gi|45382614| | -1.288218658 | Down |
| serine-protein kinase ATM (ATM) | gi|240848602| | -1.370735303 | Down |
| metalloproteinase inhibitor 4 (TIMP4) | gi|513204826| | -1.386279277 | Down |
| dachshund homolog 2 (DACH2) | gi|45382176| | -1.397506532 | Down |
| 60S ribosomal protein L27a (RPL27A) | gi|478621093| | -1.403904303 | Down |
| 60S ribosomal protein L37a (RPL37A) | gi|118405213| | -1.443200723 | Down |
| histone-lysine N-methyltransferase SUV39H2 (SUV39H2) | gi|189409111| | -1.481765019 | Down |
| growth hormone receptor precursor (GHR) | gi|47604939| | -1.505882693 | Down |
| amidophosphoribosyltransferase precursor (PPAT) | gi|52345389| | -1.592578347 | Down |
| beta-hexosaminidase subunit beta (HEXB) | gi|513229734| | -1.593231003 | Down |
| 60S ribosomal protein L30 (RPL30) | gi|56118965| | -1.661478057 | Down |
| 60S ribosomal protein L37 (RPL37) | gi|170650590| | -1.754794218 | Down |
| 24-dehydrocholesterol reductase (DHCR24) | gi|71896814| | -1.780922052 | Down |
| hypothetical protein PANDA_004337 (RPS23) | gi|513232151| | -1.835894519 | Down |
| centromere protein S (APITD1) | gi|326319978| | -1.855612753 | Down |
| muellerian-inhibiting factor precursor (AMH) | gi|49225565| | -1.957571089 | Down |
| probable ATP-dependent RNA helicase DDX4 (DDX4) | gi|45382658| | -2.034936453 | Down |
| 40S ribosomal protein S6 (RPS6) | gi|402692243| | -2.079123144 | Down |
| ciliary neurotrophic factor receptor subunit alpha precursor (CNTFR) | gi|45384301| | -2.175114111 | Down |
| transcription factor SOX-9 (SOX9) | gi|45383571| | -2.285297029 | Down |
| sodium/potassium-transporting ATPase subunit alpha-2 (ATP1A2) | gi|45382690| | -2.303932417 | Down |
| lutropin-choriogonadotropic hormone receptor precursor (LHCGR) | gi|45384387| | -2.357780254 | Down |
| inhibin/activin beta B subunit precursor (INHBB) | gi|45383927| | -3.662209758 | Down |
| nuclear receptor subfamily 5 group A member 2 (NR5A2) | gi|45384181| | -4.018994909 | Down |
| testosterone 17-beta-dehydrogenase 3 (ZNF367) | gi|513231050| | -5.619898953 | Down |
